# A longitudinal study of stavudine-associated toxicities in a large cohort of South African HIV infected subjects

**DOI:** 10.1186/1471-2334-11-244

**Published:** 2011-09-17

**Authors:** Colin N Menezes, Mhairi Maskew, Ian Sanne, Nigel J Crowther, Frederick J Raal

**Affiliations:** 1 Infectious Diseases Unit, Department of Medicine, Helen Joseph Hospital, University of the Witwatersrand, Johannesburg, South Africa; 2 Clinical HIV Research Unit, Helen Joseph Hospital, University of the Witwatersrand, Johannesburg, South Africa; 3 Themba Lethu Clinic, Helen Joseph Hospital, Johannesburg, South Africa; 4 Department of Chemical Pathology, National Health Laboratory Service and University of the Witwatersrand, Johannesburg, South Africa; 5 Division of Endocrinology & Metabolism, Department of Medicine, University of the Witwatersrand, Johannesburg, South Africa

## Abstract

**Background:**

There has been major improvement in the survival of HIV-1 infected individuals since the South African Government introduced highly active anti-retroviral therapy (HAART) in the public sector in 2004. This has brought new challenges which include the effects of stavudine-related toxicities.

**Methods:**

Prospective analysis of a cohort of 9040 HIV-infected adults who were initiated on HAART at the Themba Lethu Clinic (TLC) in Johannesburg between April 1, 2004 to December 31, 2007, and followed up until June 30, 2008.

**Results:**

Amongst the 9040 study subjects, 8497(94%) were on stavudine based therapy and 5962 (66%) were women. The median baseline CD4 count was 81 cells/mm^3 ^(IQR 29-149). Median follow up on HAART was 19 months (IQR: 9.1-31.6). The proportion of HAART-related side effects for stavudine compared to non-stavudine containing regimens were, respectively: peripheral neuropathy,17.1% vs. 11.2% (p < 0.001); symptomatic hyperlactataemia, 5.7% vs. 2.2% (p < 0.0005); lactic acidosis, 2.5 vs. 1.3% (p = 0.072); lipoatrophy, 7.3% vs. 4.6% (p < 0.05). Among those on stavudine-based regimens, incidence rates for peripheral neuropathy were 12.1 cases/100 person-years (95%CI 7.0-19.5), symptomatic hyperlactataemia 3.6 cases/100 person-years (95%CI 1.2-7.5), lactic acidosis 1.6 cases/100 person-years (95%CI 0.4-5.2) and lipoatrophy 4.6 cases/100 person-years (95%CI 2.1-9.6). Females experienced more toxicity when compared to males in terms of symptomatic hyperlactataemia (p < 0.0001), lactic acidosis (p < 0.0001), lipoatrophy (p < 0.0001) and hypertension (p < 0.05).

**Conclusions:**

We demonstrate significant morbidity associated with stavudine. These data support the latest WHO guidelines, and provide additional evidence for other resource limited HAART rollout programs considering the implementation of non-stavudine based regimens as first line therapy.

## Background

By the end of 2008, an estimated 33.4 million people worldwide were living with human immunodeficiency virus (HIV) infection [1]. Southern Africa continues to bear a disproportionate share of the global burden of HIV with 67% of the HIV infections worldwide, of which 68% of them were amongst adults. This region also accounted for 72% of the world's AIDS related deaths [1]. South Africa's 2009 HIV prevalence rate in the adult population (aged 15-49 years) was estimated to be 17.8% [2].

There has been an increase in the provision of highly active antiretroviral therapy (HAART), with up to 44% of adults and children estimated to be receiving therapy with a profound reduction in mortality [2] and, with adherence to HAART it is possible to transform HIV from a fatal infection to a chronic and manageable illness [3-5].

However, some of the anti-retroviral agents used in HAART regimens have severe side effects. Prominent amongst these drugs is stavudine, the use of which is associated with lactic acidosis/symptomatic hyperlactataemia, lipoatrophy, and peripheral neuropathy [6]. Other side effects of stavudine use include dyslipidaemia and insulin resistance, and it is an independent risk factor for the development of new onset diabetes mellitus [7]. Despite these serious side effects up to 60% of HIV-positive patients in low and middle income countries are receiving stavudine [8,9].

The aim of this study was to make use of a large (N = 9040) clinic-based survey of HIV-positive patients newly initiated onto HAART over a period of three years, to compare and describe the effect of stavudine based therapy on acute and chronic toxicities after initiation of HAART. We present baseline data gathered before the initiation of HAART and data collected for three years with each patient having a minimum of six months of follow-up. This is the largest study of the side effects of stavudine therapy conducted to date in a South African HIV-positive population.

## Methods

### Study population

The study population included HIV-1 infected individuals attending the Themba Lethu Clinic, a public sector HAART rollout facility based at the Helen Joseph Hospital, a teaching hospital attached to the University of the Witwatersrand, Johannesburg, South Africa. This clinic provides free antiretroviral therapy and other specialized services, and is one of the largest HAART rollout clinics in Africa. The program is funded by the South African National and Gauteng Departments of Health, with support from Right to Care funded by USAID and PEPFAR.

### Treatment

HAART was initiated in accordance with the 2004 South African National Antiretroviral Treatment Guidelines, which include initiation criteria of a CD4 count ≤ 200 cells/mm^3 ^or WHO stage 4 AIDS defining illness irrespective of CD4 count [10]. The first line therapy consisted of stavudine, lamuvidine and efavirenz or nevirapine; however kaletra (ritonavir/lopinavir) was used as part of the first line therapy regimen if there were contra-indications to other first line drugs [10]. Until October 2007, stavudine was dosed according to patients' body weight: 30 mg for those < 60 kg and 40 mg for those ≥ 60 kg. From October 2007, a universal 30 mg dose was introduced and 40 mg tablets of stavudine were withdrawn from the clinic. Single drug substitutions were permitted depending on the underlying clinical presentation of the patient.

### Clinical and laboratory measurements

Patients who met the criteria for initiating of HAART received adherence counseling and screening for opportunistic infections prior to initiation of therapy. A history and physical examination was performed at every visit. All patients had a baseline chest X-ray. Laboratory monitoring was performed according to the clinic protocol. Other serum biochemical tests were carried out as clinically indicated. The results of these tests were not available for analysis as the majority of patients were seen and diagnosed at other clinics where they would present for acute medical problems, and where their HAART regimen was modified. However, their diagnoses and new HAART regimens were captured for this study.

### Diagnosis and definitions

Body mass index (BMI) was defined as body weight divided by the height, squared (kg/m^2^). Patients who presented with symptoms of numbness or dysesthesia after initiation of HAART were defined as having peripheral neuropathy due to HAART, once other causes were excluded. Symptomatic hyperlactataemia was defined as the presence of suggestive symptoms with an uncuffed venous lactate level > 5 mmol/L with no evidence of a metabolic acidosis; and lactic acidosis was defined as an uncuffed lactate > 5 mmol/l and arterial pH < 7.35 or a total venous CO_2 _< 20 mmol/l, with other causes such as sepsis, renal failure, diabetic ketoacidosis and dehydration excluded. Pancreatitis was defined as the presence of abdominal symptoms with a serum amylase > 125 U/L and lipase > 60 U/L. The definition of lipoatrophy was based on the development of peripheral fat wasting (face, arms, buttocks or thighs) and/or central abdominal fat accumulation, and may include enlarged breasts. This was usually reported by the patient and confirmed by the doctor or, initially diagnosed by the doctor with patient confirmation. Hypertension was defined by the presence of three separate readings of systolic blood pressure > 140 mmHg and a diastolic blood pressure > 90 mmHg. Diabetes was defined as the presence of symptoms with a fasting glucose of > = 7 mmol/L or a random blood glucose of 11.1 mmol/L. Dyslipidaemias were defined by the presence of abnormal lipid levels which included an elevated total cholesterol of > 5 mmol/L, an elevated triglyceride level of > 1.7 mmol/L, and elevated LDL level of > 3 mmol/L.

### Data collection and statistical analysis

We analyzed prospectively collected longitudinal cohort data from patients attending the clinic. Clinical data from patient records were captured onto an electronic database via a medical management software system, Therapy Edge-HIV™ (Associated Biological Systems, South Africa). Data was analyzed using the SAS^® ^9.1 statistical software package (SAS Institute, Inc., North Carolina, USA). Baseline characteristics of the study sample were summarized using simple proportions and medians with interquartile ranges. Differences in proportions of the toxicities were compared by initiating regimen with Chi-squared tests. Study subjects were followed from HAART initiation to the earliest of 1) death; 2) loss to follow up; 3) development of toxicity or 4) censor date 31 December 2008. Incidence rates with 95% confidence intervals for toxicity were calculated and compared by initiating regimen type (stavudine-based versus other). Crude and adjusted estimates of the effect of stavudine use on development of incident toxicity were estimated using Cox proportional hazard models. Models were controlled for confounding by baseline body mass index, CD4 count, age, and gender. Though the majority of subjects initiated HAART prior to the introduction of the universal 30 mg stavudine dose, models were also adjusted for time period in which HAART was initiated (prior to or post October 2007). Kaplan Meier curves were used to estimate crude time to diagnosis of therapy-related complications stratified by initiated regimen. Subjects with existing toxicity at initiation of HAART were excluded from these analyses.

Use of the data for the study was approved by the Human Research Ethics Committee (Medical) of the University of the Witwatersrand.

## Results

**Figure 1:**
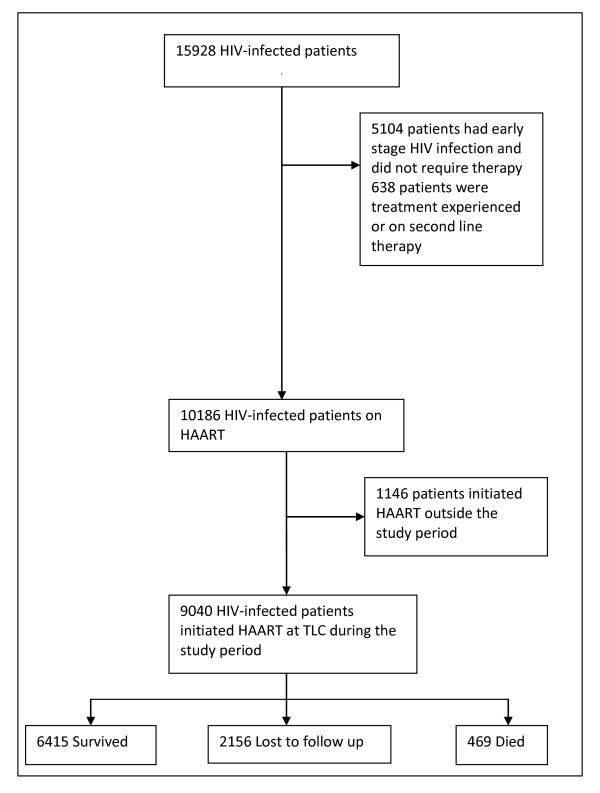
**Profile of the Study Cohort**.

Between 1 April 2004 and 1 July 2008, a total of 15,928 HIV-infected adults enrolled in care at the Themba Lethu Clinic. Of these, 5104 (32%) had early stage HIV infection and did not qualify for HAART, whilst the remaining 10,824 (68%) subjects were initiated on HAART. The study sample included the 9040 patients initiated on treatment between 1 April 2004 and 31 December 2007. The cohort profile is summarized in Figure 1.

### Baseline characteristics of the study population

The baseline characteristics of the study cohort are summarized in Table 1. Two thirds of the study group was female. The majority of patients were in the age group 25-44 years. Even though 46.9% of the total study population were defined as being at only WHO Stage 1 for AIDS, they were already on HAART, indicating that their CD4 count rates were below 200 cells/mm^3 ^and this was confirmed by the median baseline CD4 count of 81 cells/mm^3 ^(IQR: 29-149).

**Table 1:** Cohort characteristics at baseline

Characteristic	Baseline value
**Gender** Female Male	**(N = 9040)** 5962 (66) 3078 (34)

**Age (years)** < 25 years 25-34 years 35-44 years 45-54 years > 55 years	**(N = 9040)** 551 (6.1) 3822 (42.3) 3216 (35.5) 1173 (13.0) 277 (3.1)

**WHO AIDS classification** Stage 1 Stage 2 Stage 3 Stage 4	**(N = 8714)** 4086 (46.9) 1310 (15) 2507 (28.8) 811 (9.3)

**HAART** d4T/3TC/EFV d4T/3TC/NVP d4T/3TC/Kaletra AZT containing regimen TDF containing regimen other	**(N = 9040)** 7138 (79) 690 (7.6) 669 (7.4) 308 (3.4) 59 (0.7) 176 (1.9)

**BMI (kg/m^2^)**	22.4 ± 5.0 (**N = 7010)**

**CD4 count (cells/mm^3^)**	81 (29-149) **(N = 7605)**

**Follow up time on HAART (months)**	19.0 (9.1-31.6) **(N = 9040)**

Data is expressed as N (%) except for BMI (mean ± SD), CD4 count (median, IQR) and follow up time on HAART (median, IQR). WHO, World Health Organization, d4T, stavudine; 3TC, lamivudine; EFV, efavirenz; NVP, nevirapine; kaletra, ritonavir/lopinavir; AZT, zidovudine; TDF, tenofovir.

Ninety four percent of patients were on stavudine based regimens at baseline, with 79% of these initiated on stavudine, lamivudine and efavirenz as per the 2004 South African National guidelines [10], while a smaller number were initiated on zidovudine- (3.4%) and tenofovir-based regimens (0.7%).

### Retention in care

The median time to follow up for this cohort on HAART was 19 months (IQR: 9.1-31.6). At the end of the study period, a total of 6415 patients (71%) were still alive and in care, 469 were confirmed deceased and a further 2156 were considered lost to follow up. Patients were considered lost to follow up if they missed their last scheduled appointment by more than 90 days or at least 180 days had lapsed since their last visit. It can be assumed that some of the patients who are lost to follow up may have died and so in total, there were 2,625 (16.5%) patients in this cohort who were either lost to follow up or dead by the end of the study period (see Figure 1).

### Response to HAART

The patients had a median baseline CD4 count of 81 cells/mm^3 ^(IQR 29-149). There was a significant (p < 0.0001) increase in the CD4 count after initiation with a median of 205 cells/mm^3 ^(IQR 132-293) by 6 months on treatment. After six months of therapy, there was no significant change in BMI (means ± SD; 22.4 ± 5.0 and 23.7 ± 5.5 respectively).

### Acute and chronic toxicities

Amongst the 9040 patients on therapy, 2488 patients (27.5%) had one or more incident toxicities recorded after treatment initiation. In terms of acute HAART-related toxicities, the proportion of patients diagnosed with peripheral neuropathy was significantly higher in the group receiving stavudine-based therapy (17.1% vs. 11.2%; p < 0.001) compared to those on non-stavudine based therapy (Table 2). Peripheral neuropathy was reported equally in both genders (approximately 17%), with no difference in time to development between the drug groups. There was also a significantly higher proportion of patients on stavudine based therapy presenting with symptomatic hyperlactataemia when compared to those on non-stavudine based therapy (5.75 vs. 2.2%, p < 0.0005) (Table 2), with females more frequently affected than males (7.1% vs. 2.5%; p < 0.0001). Although the rate of development of lactic acidosis was the same for both drug groups, it was experienced more frequently in females than males (3.3% vs. 0.8%; p < 0.0001). The median time to onset was the same for both drug groups. Pancreatitis was equally rare in both treatment groups (see Table 2); both genders were equally affected, with the time to onset being the same in both drug groups. When compared to those on non-stavudine based regimens, those receiving stavudine had higher incidence rates of several toxicities including peripheral neuropathy [12.1/100 person-years (95%CI 7.0-19.5) vs. 7.9/100 person-years (95%CI 6.0-10.1)], symptomatic hyperlactataemia [3.6/100 person-years (95%CI 1.2-7.5) vs.1.4/100 person-years (95%CI 0.7-2.5)], and lactic acidosis [1.6/100 person-years (95%CI 0.4-5.2) vs. 0.8/100 person-years (95%CI 0.3-1.7)] (Table 3).

**Table 2:** Frequency of and time to HAART associated toxic complications by initiating regimen

Condition	Stavudine (N = 8497)	Other drugs (N = 543)
**Peripheral neuropathy**		
Frequency	1454 (17.1)*	61 (11.2)*
Time to diagnosis	6.7 (3.8-111.8)	5.1 (3.1-11.7)

**Symptomatic hyperlactataemia**		
Frequency	487 (5.7)*	12 (2.2)*
Time to diagnosis	14.5 (10.6-20.9)	11.6 (8.7-19.4)

**Lactic Acidosis**		
Frequency	214 (2.5)	7 (1.3)
Time to diagnosis	10.8 (9.0-13.5)	11.2 (9.0-13.5)

**Pancreatitis**		
Frequency	14 (0.2)	1 (0.2)
Time to diagnosis	10.4(4.0-13.0)	8.3 (8.3-8.3)

**Lipoatrophy**		
Frequency	616 (7.3)**	25 (4.6) **
Time to diagnosis	17.0 (11.4-23.1)	15.0 (10.5-24.8)

**Hypertension**		
Frequency	167 (2.0)	9.7 (4.6-18.4)**
Time to diagnosis	17 (3.1)	(9.3-30.5)**

**Diabetes**		
Frequency	28 (0.3)	1 (0.2)
Time to diagnosis	22.4 (12.5-28.1)	9.0 (9.0-9.0)

**Dyslipidaemia**		
Frequency	109 (1.3)	9(1.7)
Time to diagnosis	24.6 (17.4-34.0)	19.1 (15.3-26.1)

Data is given as n (%) for prevalence and median (IQR) for time to diagnosis;

*p < 0.005, **p < 0.05 versus group receiving stavudine.

**Table 3:** Crude and adjusted effects of stavudine use on toxicity initiated on HAART

Toxicity	No events	Person Time (years)	Rate/100 pys* (95% CI)^‡^	Crude HR^§^ (95% CI)^‡^	Adjusted† HR (95% CI)^‡^
**Peripheral Neuropathy** *Other* *d4T-based*	61 1454	775.9 12055.5	7.9 (6.0-10.1) 12.1 (7.0-19.5)	1.0 1.53 (1.19-1.98)	1.0 2.02 (1.35-3.03)

**Symptomatic hyperlactataemia** *Other* *d4T-based*	12 487	852.3 13690.9	1.4 (0.7-2.5) 3.6 (1.2-7.5)	1.0 2.70 (1.52-4.79)	1.0 2.81 (1.26-6.31)

**Lactic acidosis** ***Other*** ***d4T-based***	7 214	848.3 13805.9	0.8 (0.3-1.7) 1.6 (0.4-5.2)	1.0 2.09 (0.99-4.44)	1.0 2.55 (0.81-8.00)

**Pancreatitis** *Other* *d4T-based*	1 14	858.9 14126.2	0.1 (0.003-0.6) 0.1 (0.02-2.6)	1.0 0.95 (0.13-7.21)	1.0 0.40 (0.05-3.16)

**Lipoatrophy** *Other* *d4T-based*	25 616	834.0 13534.3	3.0 (1.9-4.4) 4.6 (2.1-9.6)	1.0 1.67 (1.12-2.50)	1.0 1.60 (0.92-2.77)

**Hypertension** *Other* *d4T-based*	17 167	845.0 13929.0	2.0 (1.2-3.2) 1.2 (0.2-4.0)	1.0 0.66 (0.40-1.08)	1.0 0.83 (0.39-1.78)

**Diabetes Mellitus** *Other* *d4T-based*	1 28	859.0 14122.7	0.1 (0.003-0.6) 0.2 (0.02-2.6)	1.0 1.90 (0.26-14.04)	1.0 2.07 (0.28-15.21)

**Dyslipidaemia** *Other* *d4T-based*	9 109	856.5 14097.2	1.1 (0.4-2.0) 0.8 (0.2-4.0)	1.0 0.82 (0.41-1.61)	1.0 0.61 (0.27-1.40)

*pys = person years

^§ ^HR = hazard ratio estimated from Cox proportional hazard models

^‡ ^95% CI = 95% confidence interval

^† ^All models adjusted for age, gender, baseline CD4 count, baseline body mass index and time at which HAART was initiated (either prior to or post October 2007)

In terms of the chronic HAART-related metabolic complications, 7.3% presented with lipoatrophy on stavudine based therapy, compared to 4.6% (p < 0.05) patients on non-stavudine based therapy. The median time to development of lipoatrophy was similar across the 2 treatment groups. Lipoatrophy was more predominantly seen in female than male patients (10.0% vs. 1.6%; p < 0.0001). Incidence rates for lipoatrophy were slightly higher in the stavudine based therapy group [3.0/100 person-years (95%CI 1.9-4.4) versus 4.6/100 person-years (95%CI 2.1-9.6)] compared to those on other regimens (Table 3). Only two percent of patients presented with hypertension, while only 0.3% presented with diabetes and 1.3% with dyslipidaemia on stavudine based therapy. Similar proportions developing these conditions were observed in both groups (Table 2). However, hypertension developed more quickly (p < 0.05) in patients taking stavudine than in those not receiving this drug (Table 2). Hypertension was less common in females than males (1.8% vs. 2.4%; p < 0.05) but females and males were equally affected in terms of diabetes and dyslipidaemia.

In multivariate analyses adjusted for age, gender, baseline BMI, CD4 counts and the time period initiating HAART, the use of stavudine continued to be associated with increased hazard of developing several toxicities: peripheral neuropathy (HR 2.02; 95% CI 1.35-3.03), symptomatic hyperlactataemia (HR 2.81; 95% CI 1.26-6.31), lactic acidosis (adjusted HR 2.55; 95% CI 0.81-8.00) and diabetes mellitus (adjusted HR 2.07; 95% CI 0.28-15.21) though some of these estimates lacked precision (Table 3).

**Figure 2:**
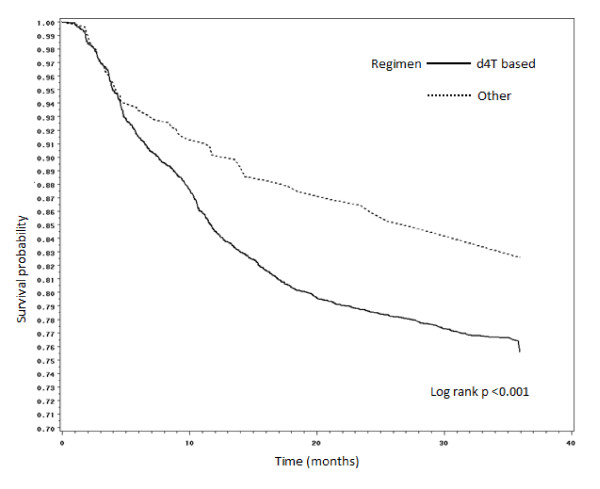
**Kaplan Meier crude estimates of time to development of peripheral neuropathy by initiating regimen (subjects with peripheral neuropathy at initiation of HAART were not included in the analysis)**.

**Figure 3:**
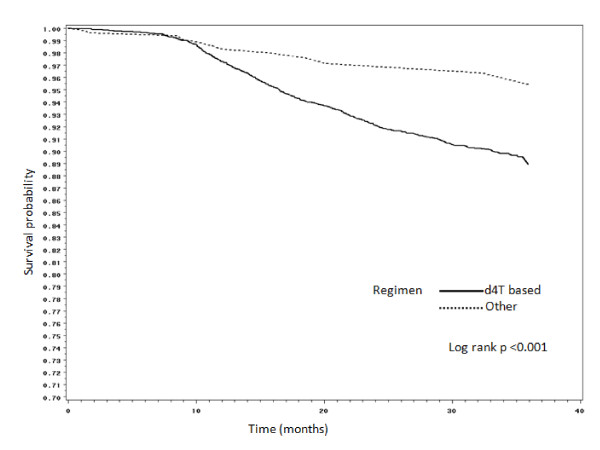
**Kaplan Meier crude estimates of time to development of hyperlactataemia by initiating regimen**.

**Figure 4:**
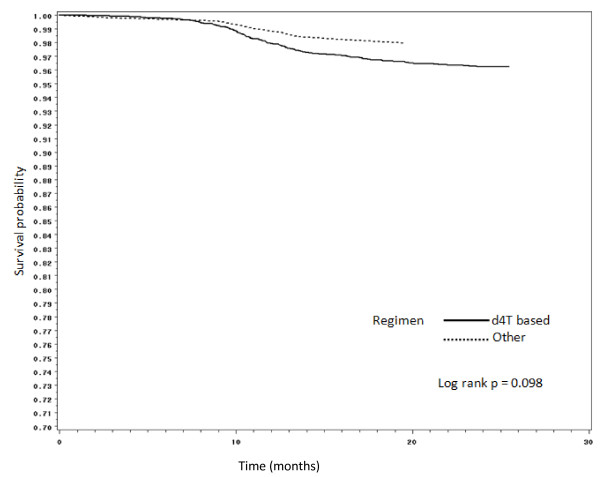
**Kaplan Meier crude estimates of time to development of lactic acidosis by initiating regimen**.

**Figure 5:**
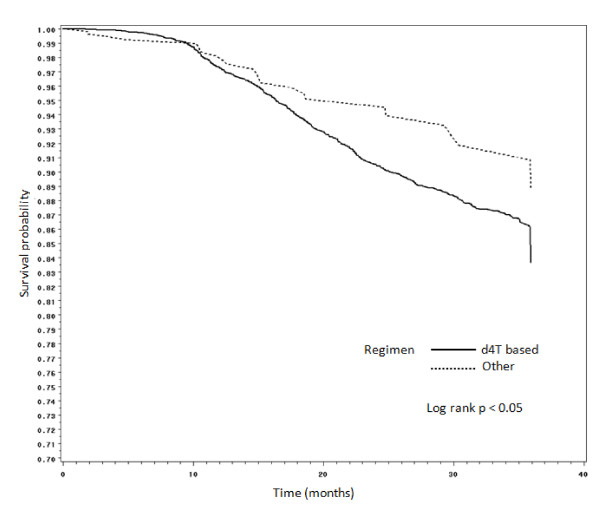
**Kaplan Meier crude estimates of time to development of lipoatrophy by initiating regimen**.

Figures 2, 3, 4 and 5 present Kaplan Meier curves for the crude estimates of the time to development of HAART-related toxicities by initiating regimen. Those initiated on stavudine based regimens were more likely to develop peripheral neuropathy (log rank p < 0.001), hyperlactataemia (log rank p < 0.001), lactic acidosis (log rank p = 0.098) or lipoatrophy (log rank p < 0.05) than those on non-stavudine based regimens.

### Other risk factors for development of toxicity

Age, CD4 counts and WHO staging at baseline did not appear to increase the risk of any adverse events after HAART initiation; however, BMIs at baseline did significantly increase the risk of both symptomatic hyperlactataemia and lactic acidosis. Relative to a baseline BMI of < 25 kg/m^2^, an increased risk of symptomatic hyperlactataemia was seen for those with a BMI of 25-30 kg/m^2 ^(RR 1.7; 95% CI 1.34-2.14, p < 0.0001) and those with a BMI of > 30 kg/m^2 ^(RR 1.82; 95% CI 1.35-2.44, p < 0.0001). The same was seen with lactic acidosis, where an increased risk was seen for those with a BMI of 25-30 kg/m^2 ^(RR 2.52; 95% CI 1.74-3.65, p < 0.0001) and those with a BMI of > 30 kg/m^2 ^(RR 3.10; 95% CI 2.00-4.79, p < 0.0001) when compared to subjects with a BMI of < 25 kg/m^2^.

## Discussion

This study describes data of three years of follow up of 9040 HIV-infected adults initiated on anti-retroviral treatment at the Themba Lethu Clinic, Johannesburg, South Africa. This high number of patients demonstrates the ability of rolling out a successful HAART programme despite being in a resource limited environment, and this, at a rapidity and scale that compares with reports from other African countries [6,11,12].

Despite the fact that stavudine is associated with significant complications, a large proportion of low and middle-income countries still use stavudine based HAART as first line therapy [8,9], mainly because of the cost implications of alternative drugs. In the present study nearly 30% of patients had to switch to non-stavudine based regimens, due to major side effects. These findings concur with those from another large study in South Africa where 21% of patients switched regimens over a similar time period [6].

This study provides estimates of the pattern of toxicities, the predominant ones being peripheral neuropathy, symptomatic hyperlactataemia, and lipoatrophy. Our incidence rates of peripheral neuropathy (12.1/100 person-years) were much higher compared to other African studies: 5.2/100 person-years in Rwanda [13] and 2.8/100 person-years in another site in South Africa [6]. The variability in these rates could be because there are no grading protocols for the severity of peripheral neuropathies. Our incidence rates of lactic acidosis were similar to a study from another province in South Africa, 1.6 versus 1.9/100 person-years [14]. The proportion of lactic acidosis was slightly higher compared to another study from Botswana [15], 2.5% versus 1% in our cohort. When compared to this same study [15], the proportion developing symptomatic hyperlactataemia were much higher in our sample (5.7% versus 2%). The median time to the development of lactic acidosis was a little later in our study when compared to another study from South Africa (10.8 months versus 7.5 months) [14].

In terms of the chronic toxicities, incidence rates of lipoatrophy were 4.6/100 person-years on stavudine based therapy and 3.0/100 person-years on the non-stavudine based therapy. One study from Rwanda [13], where patients were also on stavudine based regimens showed a similar incidence rate of lipoatrophy to that reported in the present study at 4.7/100 person-years, while one other study from South Africa [6] showed a lower incidence rate of 1.4/100 person-years. Another study from Rwanda showed a much higher proportion (34%) of patients developing lipoatrophy [16]. The reason for this wide variation in lipoatrophy rates specifically, is possibly due to different diagnostic criteria used for identifying cases. Thus, in the present study and in the studies showing low but similar rates [6,13], only cases that were severe enough to warrant a regimen change were noted. In the study showing a much higher proportion, milder cases of lipodystrophy that did not require a regimen change, were also recorded [16]. An objective case definition of lipodystrophy has been developed [17]; however, it requires access to DEXA and CT imaging technology, which is often not available in resource-limited settings. Therefore, an alternative consensus definition for the diagnosis of lipodystrophy is required for such environments.

A very small number of patients presented with diabetes (0.3%) and dyslipidaemias (1.3%). However, this could be because lipid or glucose levels were only tested when clinically suspected due to cost implications, therefore probably underestimating the true prevalence of dyslipidaemia and diabetes.

The major strength of this study is the large sample size. This cohort is similar to many other resource-limited settings where there is rapid scaling-up of comprehensive HIV care and HAART. It allowed for a relatively long duration of follow up of up to three years and a fairly high retention rate of 70%, with all clinicians working on common protocols for defining the various HAART-associated toxicities. However, despite this, these findings must be considered in the light of potential limitations. It is possible that only severe toxicities that warranted a change in regimen may have been reported and this may have led to an underestimation of the rates of some of the HAART-related toxicities, particularly lipoatrophy. Also, plasma glucose and serum lipid levels were not routinely measured and thus the true rates for glucose intolerance, diabetes and dyslipidemia were not attained. Lower reported rates of the chronic toxicities could also be related to the rates of death (5%) and loss to follow up (24%), the majority of which occurred within the first six months on treatment as described in a previous report [18].

## Conclusion

These results support the move away from stavudine based regimens towards less toxic combination regimens as advocated by the World Health Organisation [19,20]. South Africa has implemented these treatment guidelines, where tenofovir has now replaced stavudine as first line therapy. However in resource-limited countries, where staduvine is still being used because of cost implications, proper pharmacovigilance systems need to be established and alternative consensus definitions and grading protocols are required for identifying various HAART-related toxicities.

## Competing interests

The authors declare that they have no competing interests.

## Authors' contributions

Study concept and design: CNM, MM, IS. Acquisition and analysis of data: MM. Interpretation of data: CNM, MM. Drafting of manuscript: CNM. Critical revisions for important intellectual content: CNM, MM, NJC, IS, FJR. All authors read and approved the final manuscript.

## Pre-publication history

The pre-publication history for this paper can be accessed here:

http://www.biomedcentral.com/1471-2334/11/244/prepub
